# Fluorescence Lifetime Imaging Microscopy Analysis of Isolated Melanosomes

**DOI:** 10.1002/cphc.202500034

**Published:** 2025-06-15

**Authors:** Mykyta Kizilov, Sujeong Jung, Vsevolod Cheburkanov, Vladislav V. Yakovlev

**Affiliations:** ^1^ Department of Biomedical Engineering Texas A&M University College Station TX 77843 USA

**Keywords:** fluorescence lifetime imaging microscopy, laser‐induced damage, melanosomes, photochemistry, pigmentation disorders

## Abstract

Melanosomes are organelles found in a wide variety of tissues throughout the animal kingdom. They contain a variety of biological molecules, but the dominant constituent is the pigment melanin, and many functions ascribed to melanosomes, such as photoprotection, are uniquely enabled by the chemical properties and structures of the melanins they contain. In this report, fluorescence lifetime imaging microscopy (FLIM) is used, for the first time, to examine fluorescent properties of pigments in melanosomes and evaluate their time evolution upon extended laser irradiation. A relatively short‐lived component is discovered in fluorescence emission and significant changes in lifetimes upon irradiation are revealed indicating structural photoinduced changes to melanin occurring on a time scale of minutes, with observations extending up to 1 h.

## Graphical abstract

Using fast fluorescence lifetime imaging microscopy, the study tracks isolated bovine and porcine melanosomes during 60 min of 515 nm laser exposure. Two decay components lengthen progressively, while amplitude ratios shift, evidencing rapid photo‐oxidative and structural alterations of melanin. Findings inform safer laser‐based dermatological and ophthalmic therapies.

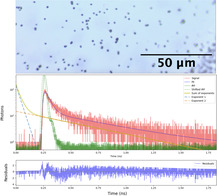

## 1 Introduction

Understanding molecular interactions within living cells is crucial for interpreting the fundamental mechanisms that underpin most cellular functions.^[^ ^1^ ^]^ In particular, the intricate interplay of these interactions not only governs signal transduction,^[^ ^2^ ^]^ gene expression,^[^ ^3^ ^]^ and protein synthesis^[^ ^4^ ^]^ but also influences the photophysical behavior of specialized organelles, such as melanosomes, which play a key role in cellular responses to light.^[^ ^5^, ^6^ ^]^ Investigating these molecular dynamics in a live‐cell context provides deeper insights into the regulatory networks that govern cellular behavior, advancing fields such as cellular biology, disease pathology, and therapeutic development.^[^ ^7^, ^8^, ^9^ ^]^

Melanosomes are specialized organelles responsible for the synthesis, storage, and transport of melanin, a critical pigment for photoprotection in the skin, eyes, and hair of mammals.^[^ ^10^ ^]^ Beyond their role in pigment production, melanosomes exhibit unique photophysical properties that directly affect light absorption and fluorescence. In retinal pigment epithelium (RPE) cells, for example, melanosomes absorb light and neutralize reactive oxygen species to protect photoreceptors from photo‐oxidative damage. Melanin exists primarily in two forms—eumelanin (a dark nitrogen‐containing polymer) and pheomelanin (a sulfur‐containing pigment with a yellowish hue);^[^ ^11^, ^12^ ^]^ variations in melanin composition can affect melanosome antioxidant capacity. Although eumelanin‐rich granules typically provide robust photoprotection,^[^ ^13^ ^]^ recent studies indicate that melanosomes can degrade when exposed to prolonged or intense light radiation.^[^ ^14^ ^]^

Despite their importance, the mechanisms underlying melanosome degradation, especially under laser or light exposure, remain poorly understood.^[^ ^12^ ^]^ Some studies suggest that melanosomes suffer irreversible photodamage,^[^ ^15^ ^]^ leading to structural disintegration and diminished antioxidant function.^[^ ^16^ ^]^ This degradation is of particular concern in clinical fields such as dermatology and ophthalmology, where lasers are used therapeutically to treat pigment‐related disorders. Understanding melanosome responses to laser exposure at a molecular level is therefore crucial for optimizing therapeutic applications and preventing adverse effects.

In most recent studies, fluorescence spectra were evaluated for melanolipofuscin granules isolated from RPE cells.^[^ ^17^ ^]^ Those results show that visible light irradiation induces oxidative degradation of melanin, leading to the formation of water‐soluble fluorescent products. Such findings provide further insight into the mechanism of light‐induced melanin loss in the RPE, emphasizing the role of superoxide radicals and oxidative stress in the deterioration of melanosomes. This work complements our observations by highlighting that similar oxidative processes may contribute to the structural and photophysical changes observed in melanosomes under prolonged laser exposure.

A variety of experimental techniques have been employed to study the morphology, structure, and chemical changes of melanosomes during light exposure.^[^ ^18^, ^19^ ^]^ Traditional microscopy methods, such as scanning electron microscopy and transmission electron microscopy, have provided insights into structural alterations. Spectroscopic methods, including Raman spectroscopy and electron paramagnetic resonance, reveal chemical changes and redox states. However, these methods can face limitations when capturing dynamic changes in live samples. Melanin's inherently low fluorescence yield further complicates analysis via standard fluorescence microscopy.

Recent advancements in laser‐based therapies have revolutionized the treatment of pigmentation disorders, providing clinicians with precise tools to target melanin‐rich tissues. Advanced imaging techniques like FLIM offer unique opportunities to study melanosome behavior in real time.^[^ ^20^ ^]^ Multispectral autofluorescence lifetime imaging, combined with machine learning, has also proven effective in diagnosing pigmented skin lesions^[^ ^21^, ^22^, ^23^ ^]^ and identifying precancerous and cancerous oral lesions.^[^ ^24^ ^]^ Nonetheless, deeper understanding of photodamage mechanisms is needed, as laser exposure may lead to melanosome fragmentation, oxidative stress, and localized heating,^[^ ^25^, ^26^ ^]^ particularly under repeated or high‐intensity applications.

Moreover, the eumelanin‐to‐pheomelanin ratio may influence the susceptibility to laser‐induced degradation.^[^ ^27^ ^]^ Identifying how structural and molecular changes emerge during laser exposure is essential to design safer therapeutic protocols. FLIM, which measures fluorescence decay times rather than intensity, is especially suited to study such processes in heterogeneous samples.^[^ ^28^, ^29^ ^]^

Short‐lifetime FLIM imaging captures ultrafast decay components often missed by conventional FLIM,^[^ ^30^, ^31^, ^32^, ^33^ ^]^ a feature relevant to studying early‐stage photodamage. By separating overlapping signals from multiple fluorophores or excited states, short‐lifetime FLIM reveals subtle molecular interactions in melanosomes.

In this study, we use FLIM to investigate melanosomes’ responses to laser exposure, tracking both short ($\text{<}10^{-1}\text{ns}$) and long ($\text{>}10^{-1}\;\text{ns}$) lifetime fluorescence components. Our aim is to explore the potential mechanisms of melanosome degradation by evaluating changes in the fluorescence lifetimes over time. Specifically, we address how laser exposure alters the molecular environment, whether distinct degradation stages can be identified from lifetime data, and the potential clinical implications for laser‐based therapies.

We employed a custom‐built confocal FLIM system with time‐correlated single‐photon counting (TCSPC) and reconvolution fitting to extract decay constants with high precision.^[^ ^34^ ^]^ These measurements enabled us to uncover subtle photophysical changes that occur under laser exposure, offering unprecedented detail on melanosome degradation dynamics.

By correlating lifetime patterns with stages of degradation, this work provides a framework for optimizing laser parameters to minimize tissue damage. Our findings may also inform research on pigment‐related diseases such as macular degeneration and melanoma,^[^ ^35^ ^]^ where melanosome behavior is key to developing effective diagnostic and therapeutic strategies.

In summary, we present a detailed FLIM‐based investigation into the photophysical behavior of melanosomes under laser exposure, highlighting short‐lifetime fluorescence components. Our results reveal a range of degradation mechanisms that underscore the promise of FLIM in advancing research on melanosome biology, dermatology, and biomedical optics.^[^ ^36^ ^]^

Recent advancements in laser‐based therapies have revolutionized the treatment of pigmentation disorders, providing clinicians with precise tools to target melanin‐rich tissues.^[^ ^37^, ^38^, ^39^ ^]^ Although techniques such as time‐resolved fluorescence spectroscopy and Raman spectroscopy^[^ ^40^, ^41^, ^42^, ^43^ ^]^ have been employed to study melanosome properties, they exhibit inherent limitations. Time‐resolved fluorescence spectroscopy generally lacks the spatial resolution needed to resolve individual melanosomes and their heterogeneous behavior, while Raman spectroscopy, despite its molecular specificity, often requires long acquisition times and is prone to interference from fluorescence background.^[^ ^42^ ^]^

Recent in vivo imaging studies have provided critical insights into the clinical significance of pigment granule alterations in the RPE. For instance, Meleppat et al.^[^ ^44^ ^]^ demonstrated that multimodal retinal imaging techniques, combining directional back‐scattering and short‐wavelength fundus autofluorescence, can effectively capture disease‐related changes in melanosome and lipofuscin densities, serving as important biomarkers for conditions such as Stargardt disease and age‐related macular degeneration. Complementarily, Dontsov and Ostrovsky^[^ ^45^ ^]^ investigated the norms and age‐related alterations of RPE pigment granules, elucidating the mechanism of light‐induced oxidative degradation that leads to a decline in melanin content. These findings underscore the potential of advanced optical imaging to reveal subtle changes in pigment composition, thereby reinforcing the relevance of our FLIM approach to studying melanosome degradation dynamics.

FLIM uniquely combines high temporal resolution with spatial mapping capabilities, allowing real‐time visualization of fluorescence decay at the single‐organelle level.^[^ ^46^, ^47^ ^]^ This dual capability not only captures rapid photophysical changes but also reveals local variations in the microenvironment—features that are critical when studying the dynamic processes of melanosome degradation under laser exposure.^[^ ^48^ ^]^ Consequently, FLIM offers a more comprehensive insight into both the kinetic and spatial aspects of melanosome photophysics compared to traditional methods.^[^ ^46^, ^47^ ^]^

## 2 Experimental Section

**Figure 1:**
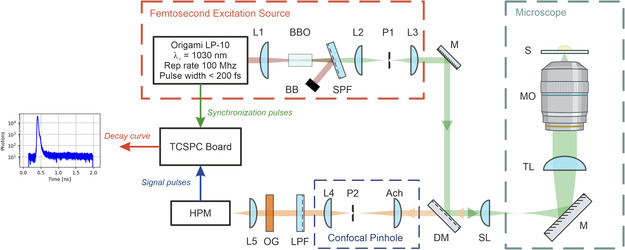
Schematic diagram of the experimental setup for fast FLIM acquisition. The setup includes a femtosecond excitation source, optical components for beam shaping and filtering, a home‐built inverted microscope with a confocal pinhole attachment, and a detection system using a hybrid photomultiplier (B&H HPM‐100‐06). Key components are labeled for clarity. BBO, beta‐barium borate SHG crystal (θ=23.4°); BB, beam block; SPF, short‐pass filter; L, plano‐convex lens; P, precision pinhole aperture; M, mirror; SL, achromatic doublet scan lens, 50 mm focal length; TL, achromatic doublet tube lens, 200 mm focal length; MO, microscope objective lens; S, sample; HPM, hybrid photomultiplier; OG, orange glass spectral filter; LPF, long‐pass filter; Ach, achromatic lens; DM, dichroic mirror. Figure made with assets created with BioRender.com.

A schematic representation of the experimental setup is depicted in **Figure** **1**.

The output from a femtosecond laser (Onefive Origami LP 10‐100) was focused onto a β‐barium borate (BBO, EKSMA Optics) crystal (cut angle θ=23.4°) using a plano‐convex lens (L1, Thorlabs, 75 mm focal length), generating second harmonic light at a wavelength of 515 nm. With an initial input power of 180 mW at 1030 nm, we achieved a conversion efficiency of 2%, producing ≈4 mW of 515 nm light. Melanosomes are characterized by a broadband, almost featureless absorption spectrum which extends to the near‐UR and UV parts of the spectrum^[^ ^49^ ^]^ and a broad fluorescence emission spectrum.^[^ ^50^ ^]^ We chose 515 nm, which happens just at the edge of a steep rise of an optical absorption while being off the peak of the fluorescence.

A hard‐coated short‐pass filter (SPF, Thorlabs, 600 nm cutoff) was used to remove the pump radiation. The spatial profile of the beam was cleaned by focusing into precision 75 μm pinhole aperture (P1, National Aperture) with a plano‐convex lens (L2, Thorlabs, 50 mm focal length). The output of the pinhole followed Airy distribution with transmitted power under 2 mW. The beam was subsequently collimated using a plano‐convex lens (L3, Thorlabs, 100 mm focal length) and directed to the custom‐built microscope via a dichroic mirror (DM, Semrock, long‐pass filter with 550 nm cutoff wavelength).

For optimal spatial resolution in the detection path, the excitation beam passed through a fixed angular magnification telescope, comprising an achromatic doublet (SL, Thorlabs, 50 mm focal length) and an achromatic doublet tube lens (TL, Thorlabs, 200 mm focal length), which expanded the beam to overfill the entrance pupil of the micriscope objective lens (MO, Nikon CFI60 series, 20×, 0.5 NA). Fluorescence emission was collected by the same objective used for its excitation. The emitted fluorescence was passed through the DM and directed to the confocal pinhole assembly.

To suppress out‐of‐focus light, the emitted signal was focused into a pinhole aperture (P2, National Aperture) with an achromatic doublet (Ach, Thorlabs, 40 mm focal length). Signal from the pinhole was collimated by a plano‐convex lens (L4, Thorlabs, 35 mm focal length) to minimize distortions caused by the necessary spectral filters. Residual excitation light is filtered by a combination of a long‐pass filter (LPF, Semrock, OD > 4.0 at 515 nm) and an orange glass filter (OG, Thorlabs, OD > 5.0 at 515 nm). Overall optical density (OD) of ≈7.0 provided sufficient attenuation of unwanted spectral components, given a laser repetition rate of 100 MHz and a maximum signal flux of $10^{7}$ photons per second. Filtered signal was then focused onto the entrance window of a hybrid photomultiplier (HPM‐100‐06, Becker & Hickl) with a plano‐convex lens (L5, Thorlabs, 75 mm focal length).

Signal from the HPM along with the synchronization pulse train from the laser was sent to the correlator (TCSPC Board) to retrieve the fluorescence decay curve, as shown in Figure 1.

The excitation power was set to 0.35 mW, ensuring sufficient signal strength while maintaining stable fluorescence lifetime measurements. While laser‐induced degradation of melanosomes is an important consideration, optimizing laser power to systematically study its effects on degradation was beyond the scope of this study. Future investigations will explore the dependence of melanosome degradation on laser power.

## 3 Sample Preparation and Experimental Design

### 3.1 Sample Preparation

The preparation procedure of bovine and porcine pigment granules (melanosomes) followed the method of Dontsov et al.^[^ ^51^, ^52^ ^]^ This method provided samples enriched in light and heavy fractions of melanosomes by density for both bovine and porcine samples. Light melanosomes are predominantly spherical, while the heavy melanosomes are predominantly elliptical in shape, resulting in slight size differences between the two fractions.^[^ ^53^ ^]^ RPE pigment granules stimulate the photo‐oxidation of unsaturated fatty acids.^[^ ^51^ ^]^ However, no high‐resolution imaging was used, and therefore, no precise knowledge of shape variance within each fraction was obtained. The melanosome fractions were separated, and stock solutions of the melanosomes were stored at 4 °C. Several dilutions of melanosomes were prepared using deionized water for each fraction prior to analysis. Plated aqueous melanosomes were prepared on glass microscope slides in order to observe cavitation events. A loosely sealed silicone washer and glass cover slip were used to enclose the melanosome sample to prevent evaporation of the aqueous suspension.

TCSPC was employed for data acquisition. Each acquisition session lasted 60 cycles of 1 min, with fluorescence decay curves recorded over a 2 ns window, producing a channel resolution of ≈500 fs.

### 3.2 Retrieval of the Instrument Response Function

The system's detectors exhibit an instrument response function (IRF) with a pulse duration width at the half maximum (commonly referred to as the full width at half maximum, FWHM) of ≈20 ps, allowing for the observation of relaxation phenomena on picosecond timescales. To ensure accurate extraction of fluorescence lifetimes, it is essential to obtain the true IRF for each acquisition rather than relying on a simulated one.

The IRF was measured by removing the OG filter and placing a silver‐coated mirror on the sample stage in the experimental setup (Figure 1). The axial position of the microscope objective was adjusted to maximize the reflection signal detected by the HPM. The detected signal, consisting of the laser pulses reflected from the mirror surface, provided the necessary data to retrieve the system's IRF.

### 3.3 Imaging Protocol

To prepare samples for imaging, 5 μL of melanosome solution was pipetted onto a concave microscope slide (Cole‐Parmer) and sealed with a glass coverslip (VWR International) to prevent exposure to air and maintain sample integrity.

Before imaging, the system was aligned using a short‐lifetime fluorophore (4‐[4‐(dimethylamino)styryl]‐1‐methylpyridinium iodide (4‐DASPI, MilliporeSigma) in water), which served as an alignment marker. A 25 μL aliquot of 4‐DASPI solution was placed on a concave slide and covered with a glass coverslip. The sample was positioned in the focal plane of the microscope objective, and adjustments were made along the optical axis to maximize the detected fluorescence signal.

After alignment, a blank slide and coverslip were inserted to confirm the absence of detectable photons, ensuring any detected signals were exclusively from the sample of interest. This validation confirmed proper alignment and preparation of the optical system for the subsequent experiment.

**Figure 2:**
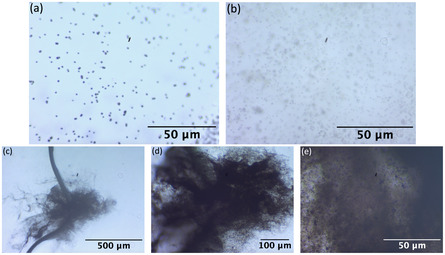
Optical microscope images of melanosome organelles observed under different magnifications. a) Planar sample of unaggregated melanosomes showing uniform distribution observed under 40× magnification. b) Volumetric distribution of unaggregated melanosomes, where overlapping regions result in a blurred appearance due to depth‐of‐field limitations observed under 40× magnification. c) 4× magnification reveals large clusters of aggregated melanosomes. d) 10× magnification provides a closer view of the aggregation patterns. e) 40× magnification shows detailed structures within the aggregates, indicating possible interactions or binding between organelles.

During the microscopy analysis, we made several interesting visual observations regarding the spatial arrangement and behavior of melanosomes. In some cases, we noticed that melanosomes tended to cluster together (**Figure** **2**c,d,e), forming tight groups or aggregates. This clustering behavior may indicate interactions between melanosomes or local changes in the sample environment.

At other times, melanosomes were more uniformly distributed across the sample (Figure 2a,b), appearing as individual particles separated from one another. This homogeneous separation suggests a more stable dispersion, potentially influenced by the preparation process or environmental factors. When the concentration of melanosomes was high, multiple layers formed, and focusing on one layer resulted in melanosomes in other layers appearing blurred.

**Figure 3:**
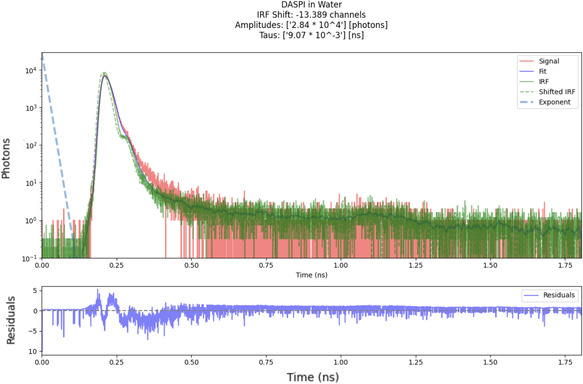
Reconvolution fit of the TCSPC data for 4‐DASPI in water. The red line represents the signal, the green line is the IRF, and the blue line is the reconvolution fit.

Additionally, in some observations, melanosomes appeared to be connected by a viscous liquid (Figure 2, 3), forming a network‐like structure. This could be due to residual components in the sample, such as proteins or lipids, that form a gel‐like matrix surrounding the melanosomes. These variations in melanosome behavior under the microscope provide valuable context for understanding their dynamic nature and interactions during laser exposure.

## 4 Obtaining Decay Constants through Reconvolution Fit

The Savitzky–Golay smoothing filter^[^ ^54^ ^]^ is a widely used technique for smoothing a set of data points, effectively preserving the shape and features of the signal, such as peaks and valleys, better than other averaging filters. This filter works by fitting successive subsets of adjacent data points with a low‐degree polynomial through the method of linear least squares. Savitzky–Golay filter was applied to all collected Signal and IRF before analyzing.

### 4.1 Reconvolution Fitting Process

The reconvolution fitting process is a crucial step in analyzing TCSPC data. The goal is to model the observed fluorescence decay curve by deconvolving the IRF from the measured signal, allowing accurate estimation of the decay parameters of the sample.^[^ ^55^ ^]^

Reconvolution fitting is essential for accurately extracting decay parameters from TCSPC data. In this approach, the measured signal S(t) is modeled as the convolution of the sample's actual fluorescence decay F(t) and the instrument response function R(t), i.e., $S(t)=\int_{0}^{\infty }F(t\prime )\;R(t-t\prime )\;dt\prime$. The decay is typically represented as a sum of exponentials, $F(t)=\sum_{i=1}^{n}A_{i}e^{-t/\tau_{i}}$, and the IRF^[^ ^56^ ^]^ is aligned using a noninteger shift if necessary. To find the best‐fit parameters (decay constants $\tau_{i}$, amplitudes $A_{i}$, and IRF shift Δt), we employ a global optimization (e.g., Differential Evolution),^[^ ^57^ ^]^ followed by non‐negative least squares (NNLS). This process ensures robust fitting of the observed TCSPC data and allows for precise determination of fluorescence lifetimes.

### 4.2 Fit Validation with 4‐DASPI

To validate the reconvolution fitting process, we used 4‐DASPI dissolved in water as a standard reference. 4‐DASPI is known for its short fluorescence lifetime in water at around 10.^[^ ^58^, ^59^ ^]^

We performed a TCSPC measurement on a 25 μL sample of 4‐DASPI diluted in water under the same experimental conditions as for the melanosome measurements. The fluorescence decay curve was fitted using the same reconvolution procedure, where the IRF was measured in real time.

This validation step provides confidence in the fitting procedure and ensures that the decay times for melanosome fluorescence lifetimes can be accurately retrieved (**Figure** **3**).

## 5 Data Analysis

We continuously imaged melanosomes for 60 min at 1 min intervals under 0.35 mW laser exposure. We specifically focused on melanosomes that were immobilized between the concave slide and coverslip, minimizing movement due to Brownian motion.^[^ ^60^ ^]^ Throughout the imaging process, changes in the fluorescence signal were observed, including variations in intensity and peak shape, which reflect dynamic shifts in both fluorescence intensity and lifetime.

To reduce the impact of Brownian motion on fluorescence intensity measurements, we allowed the sample to partially dry before imaging, reducing mobility. Additionally, the excitation volume was relatively large compared to individual melanosomes, mitigating signal fluctuations caused by minor displacements. We selected the most immobilized melanosomes for analysis and confirmed their position remained stable within the field of view throughout the experiment.

**Figure 4:**
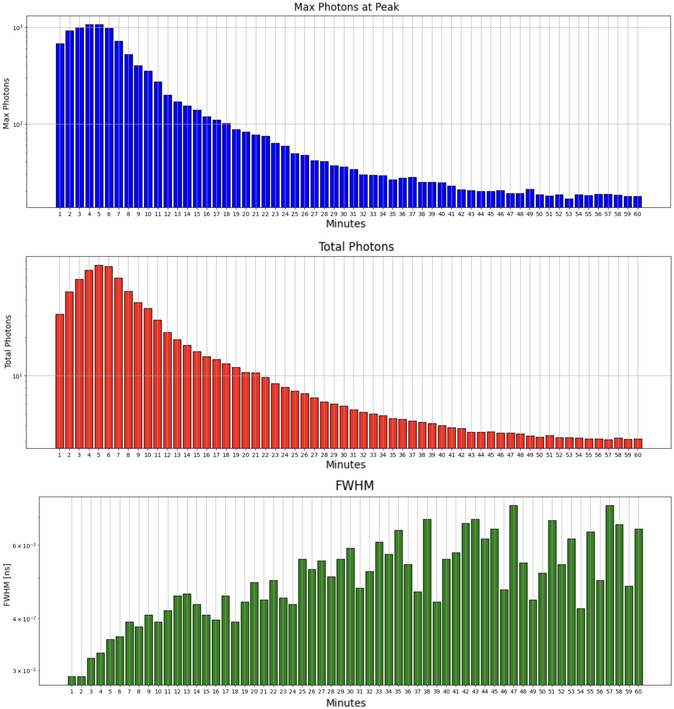
Initial rise followed by a decline. Top: Peak photon counts per minute. Middle: Total photons detected per minute. Bottom: FWHM of spectra per minute.

During continuous imaging of melanosomes over 60 min, we identified multiple patterns in their fluorescence response, including random variations likely caused by Brownian motion,^[^ ^60^ ^]^ gradual or monotonic changes in fluorescence parameters, and complex combinations of multiple processes. However, in this study, we focus on the most prominent pattern observed. This rise‐and‐fall pattern, depicted in **Figure** **4**, can be attributed to several causes. Laser‐induced activation and degradation may explain the phenomenon, with initial fluorescence increase due to enhanced molecular excitation, and subsequent decline due to degradation or molecular rearrangement. Photobleaching from continuous laser exposure might cause gradual loss of fluorescence intensity. Additionally, thermal effects could initially boost fluorescence, later decreasing due to molecular denaturation or damage. The implications of this pattern highlight a combination of activation and degradation processes, illustrating the dynamic nature of melanosome response to laser exposure.

It is worth noting that even when fluorescence variation might be the same, total intensity might still be different.^[^ ^61^ ^]^ We can observe this discrepancy mainly because of Brownian motion. Despite the fact that we tried to aim at the most “still” melanosomes, we did not completely eliminate this effect.

For each recording that we have, we performed reconvolution fit to extract decay constants.

In Figure 4, we can observe the evolution of key parameters during imaging. The total number of detected photons, peak photon counts, and the FWHM of the fluorescence spectra are plotted for each minute of imaging. While the total and peak photon counts exhibit an initial rise followed by a decline, the FWHM continues to increase over time. This suggests that distinct photophysical processes influence spectral broadening and intensity changes in melanosomes during prolonged laser exposure.

**Figure 5:**
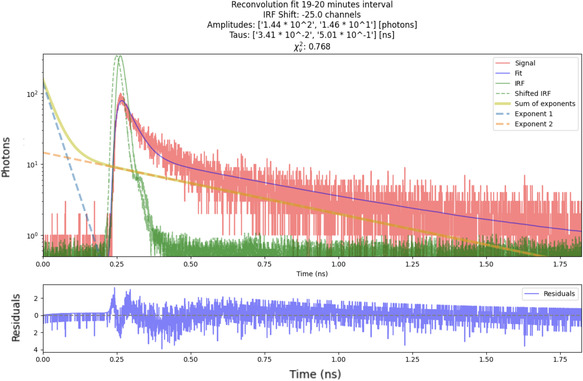
Reconvolution fit of the fluorescence decay data for melanosomes after 20 min of laser exposure, corresponding to the data in Figure 4. The measured decay curve (dots) and the fitted model (solid line) are shown, illustrating the extraction of decay constants $\tau_{1}$ and $\tau_{2}$. Residuals indicate the goodness of fit achieved by the reconvolution method.

To extract the fluorescence lifetimes, we performed a reconvolution fitting procedure on the fluorescence decay curves. A sample fit is presented in **Figure** **5**, showing the quality of the reconvolution for a 19–20 min interval. The fitting process allowed us to extract two lifetime components, $\tau_{1}$ and $\tau_{2}$, which describe the fast and slow decay processes, respectively.

**Figure 6:**
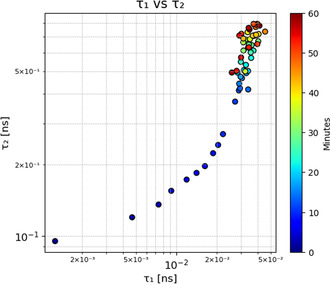
Evolution of the fluorescence lifetime components $\tau_{1}$ (fast decay) and $\tau_{2}$ (slow decay) over 60 min of continuous laser exposure, corresponding to the data in Figure 4. Both lifetime components increase steadily over time, suggesting structural or chemical changes in melanosomes due to laser‐induced effects.

As shown in **Figure** **6**, both lifetime components, $\tau_{1}$ and $\tau_{2}$, increased steadily over the course of the 60 min of laser exposure. Notably, the variability in lifetime estimates becomes more pronounced for lifetimes greater than 0.03 ns. This increased scattering for longer lifetimes is likely due to the difficulty in accurately estimating slow decay components with fast FLIM acquisition. The estimated uncertainty in extracted lifetime values is approximately ±10% for a single measurement.

To support our interpretation of fluorescence dynamics, the full set of decay constants ($\tau_{1}$, $\tau_{1}$) and their corresponding amplitudes ($A_{1}$, $A_{2}$) extracted from reconvolution fits are provided in Supplementary Table 1. This table includes numerical values for all 60 time points corresponding to Figure 6, allowing detailed inspection of trends and fitting robustness.

It should be noted that the $\chi^{2}$ values obtained from the reconvolution fitting are consistently below 1 (e.g., 0.768 in 5). This is primarily due to the application of the Savitzky–Golay filter, which effectively smooths the data and reduces noise, thereby lowering the $\chi^{2}$ value. We emphasize that this low $\chi^{2}$ reflects the high quality of our fits, which are consistent across all measurements. Potential sources of deviation may stem from differences in the probing geometry; the IRF is measured using a reflective geometry on a glass slide, whereas the melanosome sample, being volumetric, is probed using the full numerical aperture of the objective. Such geometric differences can lead to subtle variations in the extracted lifetimes.

**Figure 7:**
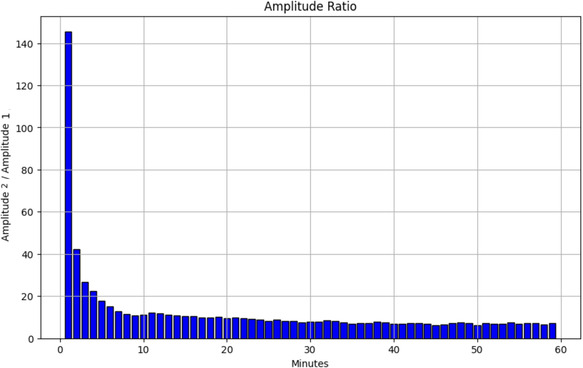
Temporal evolution of the amplitude ratio (A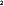/A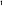) between the fast and slow decay components over 60 min of laser exposure, indicating changes in the relative contributions of the two processes. The initial rapid decrease suggests a shift in fluorescence dynamics.

The ratio of the amplitudes corresponding to the two lifetime components is plotted in **Figure** **7**. Initially, the amplitude ratio decreases rapidly within the first 10 min of imaging, after which it stabilizes and remains relatively constant for the remainder of the experiment.

After performing reconvolution fitting for 40 FLIM measurements over 60 min, we extracted the decay constants ($\tau_{1}$ and $\tau_{2}$) for each melanosome sample. The extracted decay parameters revealed a notable correlation between the two time constants, which is visualized in Figure 6.

The extracted fluorescence decay components, $\tau_{1}$ and $\tau_{2}$, represent two prominent exponential decay processes in melanosomes. However, we currently do not have direct evidence linking these lifetimes to specific degradation pathways such as oxidation or polymer rearrangement. Instead, the presence of two distinct lifetime components suggests heterogeneity in the fluorescent states or molecular environments within melanosomes. Further studies will be needed to directly correlate these lifetimes with known biochemical transformations in melanin.

To further investigate the behavior of melanosomes, we applied a robust fitting model using a second‐order polynomial with Huber loss minimization. This allowed us to more accurately characterize the relationship between the fast ($\tau_{1}$) and slow ($\tau_{2}$) decay processes.

The analysis of the amplitude ratios provided additional insights into the fluorescence behavior of melanosomes over time. The largest amplitude ratios were observed in regions with the longest decay constants, suggesting that prolonged laser exposure leads to more prominent slow‐decay components in the melanosome fluorescence.

The observed trend further emphasizes that melanosomes undergo cumulative photodamage during prolonged laser exposure, which may compromise their photoprotective function. This is especially significant for clinical applications involving laser‐based therapies, where precise control of exposure parameters is crucial to minimize unintended tissue damage. Future studies could benefit from expanding the range of laser power levels to identify specific thresholds for melanosome degradation. Furthermore, incorporating multiwavelength FLIM imaging could differentiate between eumelanin‐ and pheomelanin‐dominant melanosomes, offering deeper insights into pigment‐specific degradation dynamics.

## 6 Discussion

Photophysics and photochemistry of melanins have been the subject of extensive research; however, relatively limited efforts have been devoted to studies of melanosomes due to their structural and chemical complexity. In this report, we employ fast FLIM^[^ ^62^, ^63^, ^64^ ^]^ to provide novel insights into structural changes in melanosomes under laser exposure. FLIM is uniquely capable of sensing the local nanoenvironment and is relatively unaffected by photobleaching. Both short‐ and long‐lifetime components of fluorescence emission were recorded, reflecting the complexity of molecular processes. The complex changes of fluorescence decays were observed over minutes of laser irradiation, signifying the initiation of multiple processes such as melanin oxidation, polymer reorganization, and local structure changes that are triggered by the laser excitation.

The initial increase observed in fluorescence intensity^[^ ^65^ ^]^ indicates that laser‐induced activation of selected melanin substructures could transiently increase fluorescence output. As exposure continues, partial photobleaching probably takes place, whereas local heating might result in self‐annealing of local structural defects, which initially promoted a short‐lived fluorescence.

While photobleaching could contribute to the observed changes in fluorescence lifetime, the steady increase in both lifetime components over time suggests an underlying structural or chemical transformation rather than simple fluorophore depletion. At longer radiation exposures, gradual and steady changes in fluorescence lifetimes are likely the result of progressive oxidative modifications of melanin.

In evaluating the complex nature of FLIM dynamics upon laser irradiation, we developed a novel reconvolution software tool to facilitate robust analysis of FLIM data. By automating the process of deconvolving the instrument response function from measured fluorescence decay curves, this tool streamlines the extraction of fluorescence lifetime components across diverse experimental conditions. This software is openly accessible on GitHub,^[^ ^66^ ^]^ enabling wider adoption of advanced reconvolution‐based methods for both research and clinical applications.

These observations are supported by amplitude ratios (A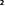/A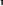) and the evolution of fast ($\tau_{1}$) and slow ($\tau_{2}$) decay constants. In many samples, an early shift toward higher A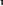 suggests that fast‐decay pathways momentarily dominate during initial laser exposure. As exposure lengthens, a higher fraction of slow‐decay components emerges, suggesting the accumulation of photoproducts, cross‐linked regions, or other reorganized melanin motifs. This interpretation aligns with prior work,^[^ ^67^, ^68^ ^]^ indicating that longer lived fluorescent states often arise from either trapped excited states or more rigid environments. Notably, temperature‐induced changes and oxidative stress can stabilize these states, increasing fluorescence lifetimes beyond initial baseline levels.

Some limitations of our experimental design include variability associated with Brownian motion, which impeded fluorescence signal stability despite attempts to immobilize the melanosomes. Additionally, the current experimental setup was limited to a single laser power and wavelength, constraining the scope of our analysis.

To overcome these limitations, optical trapping or microfluidic devices could limit sample movement and increase data consistency, while multispectral FLIM studies, varying laser power and wavelengths, would differentiate photophysical and photochemical effects more clearly. Multispectral FLIM could also distinguish between eumelanin and pheomelanin, providing further insights into pigment‐specific behavior under laser exposure.^[^ ^69^, ^70^ ^]^ FLIM can be adapted for in vivo imaging of melanosomes in tissue by leveraging its ability to measure the fluorescence decay times of naturally occurring fluorescent molecules within the skin. This technique, when combined with multiphoton laser tomography, allows for noninvasive imaging with subcellular resolution, which is crucial for detailed examination of melanosomes. In the context of melanoma diagnostics, FLIM can differentiate between melanoma and other skin lesions by identifying atypical short lifetime cells and architectural disorder, which are characteristic of melanoma, as opposed to the regular histoarchitecture seen in nevi. This capability enhances diagnostic accuracy, achieving high sensitivity and specificity, and could potentially reduce unnecessary surgical procedures by providing a more precise noninvasive diagnostic tool.^[^ ^71^ ^]^

## 7 Conclusion

This study successfully demonstrated the application of FLIM to investigate the degradation dynamics of melanosomes under laser exposure. By analyzing fluorescence lifetime components, both short‐ and long‐lived, we identified distinct photophysical changes that correlate with structural and biochemical alterations in melanosomes. The observed trends, such as the increase in fluorescence decay times and variations in amplitude ratios, strongly indicate oxidative modifications and thermal denaturation as the primary contributors to laser‐induced degradation. These findings align with previous studies highlighting the utility of FLIM for capturing dynamic cellular processes^[^ ^72^ ^]^ and monitoring molecular changes in real time.^[^ ^73^ ^]^

The heterogeneity in fluorescence patterns reflects the complex interplay of molecular excitation, photobleaching, and chemical reactions, emphasizing the need for advanced imaging techniques to resolve these dynamics. The insights provided by this study are crucial for optimizing laser parameters in clinical applications, particularly in dermatology and ophthalmology, to minimize tissue damage while achieving therapeutic goals.^[^ ^74^ ^]^

Notable novelties introduced by this study include the direct visualization and quantification of melanosome degradation in real time, enhancing our understanding of photodamage at the organelle level. Additionally, we detected a picosecond‐scale decay component, offering new insights into ultrafast photophysical dynamics associated with melanin‐containing structures.

In summary, fast FLIM is minimally invasive and provides dynamic information about structural changes in melanosomes on a time scale from minutes to hours. This study bridges gaps in understanding photodamage mechanisms and paves the way for safer and more effective laser‐based therapies. The findings presented lay the foundation for future explorations of FLIM in clinical and biomedical research, enabling real‐time monitoring of molecular interactions and dynamic processes in living tissues.

## Conflict of Interest

The authors declare no conflict of interest.

## Supporting information

Supplementary Material

## Data Availability

The data that support the findings of this study are available from the corresponding author upon reasonable request.

## References

[1] R. N. Day , F. Schaufele , Mol. Endocrinol. 2005, 19, 1675. 15761028 10.1210/me.2005-0028 PMC2900770

[2] B. D. Gomperts , P. E. Tatham , Signal Transduction, Academic Press 2009.

[3] J. H. Kang , J.‐K. Chung , J. Nucl Med. 2008, 49, 164S. 18523072 10.2967/jnumed.107.045955

[4] J. Liu , Y. Xu , D. Stoleru , A. Salic , Proc. Natl. Acad. Sci. 2012, 109, 413. 22160674 10.1073/pnas.1111561108 PMC3258597

[5] D. House , M. L. Walker , Z. Wu , J. Y. Wong , M. Betke , in 2009 IEEE Computer Society Conf. on Computer Vision and Pattern Recognition Workshops, IEEE 2009, pp. 186–193.

[6] Z. Meng , S. C. Bustamante Lopez , K. E. Meissner , V. V. Yakovlev , J. Biophotonics 2016, 9, 201. 26929086 10.1002/jbio.201500163

[7] Z. Liu , L. D. Lavis , E. Betzig , Mol. Cell 2015, 58, 644. 26000849 10.1016/j.molcel.2015.02.033

[8] V. Cheburkanov , M. Kizilov , S. Jung , M. Y. Berezin , V. V. Yakovlev , in Optical Elastography and Tissue Biomechanics XII, Vol. 13321, International Society for Optics and Photonics (Eds: K. V. Larin , G. Scarcelli ), SPIE 2025, p. 33210H.

[9] V. Cheburkanov , M. Kizilov , S. Jung , K. I. O. Sandoval , S. Raghavan , V. Yakovlev , in Label‐free Biomedical Imaging and Sensing (LBIS) 2025, Vol. 13331, International Society for Optics and Photonics (Eds: N. T. Shaked , O. Hayden ) SPIE 2025, p. 1333106.

[10] J. M. Burke , L. M. Hjelmeland , Mol. Interventions 2005, 5, 241. 10.1124/mi.5.4.7 16123538

[11] G. Prota , Melanins and Melanogenesis, Academic Press 2012.

[12] R. J. Miltenberger , K. Wakamatsu , S. Ito , R. P. Woychik , L. B. Russell , E. J. Michaud , Genetics 2002, 160, 659. 11861569 10.1093/genetics/160.2.659 PMC1461996

[13] T. Sarna , J. Photochem. Photobiol. B 1992, 12, 215. 1635010 10.1016/1011-1344(92)85027-r

[14] H. Z. Hill , W. Li , P. Xin , D. L. Mitchell , Pigm. Cell Res. 1997, 10, 158. 10.1111/j.1600-0749.1997.tb00478.x 9266603

[15] S. R. Alam , H. Wallrabe , K. G. Christopher , K. Siller , A. Periasamy , SPIE Proc. 2022, 11965, 119650B.

[16] M. Zareba , G. Szewczyk , T. Sarna , L. Hong , J. D. Simon , M. M. Henry , J. M. Burke , Photochem. Photobiol. 2006, 82, 1024. 17205626 10.1562/2006-03-08-ra-836

[17] A. E. Dontsov , M. A. Yakovleva , A. A. Vasin , A. A. Gulin , A. V. Aybush , V. A. Nadtochenko , M. A. Ostrovsky , Int. J. Mol. Sci. 2023, 24, 13099. 37685907 10.3390/ijms241713099 PMC10487480

[18] K. Jimbow , M. Jimbow , M. Chiba , J. Invest. Dermatol. 1982, 78, 76. 7054310 10.1111/1523-1747.ep12497959

[19] P. Lea , H. Haberman , A. Pawlowski , I. Menon , J. Anat. 1976, 121, 1. 1254524 PMC1231814

[20] M. J. Ruedas‐Rama , J. M. Alvarez‐Pez , L. Crovetto , J. M. Paredes , A. Orte , Advanced Photon Counting: Applications, Methods, Instrumentation 2015, pp. 191–223.

[21] P. Vasanthakumari , R. A. Romano , R. G. Rosa , A. G. Salvio , V. Yakovlev , C. Kurachi , J. M. Hirshburg , J. A. Jo , J. Biomed. Opt. 2022, 27, 066002. 35701871 10.1117/1.JBO.27.6.066002 PMC9196925

[22] P. Vasanthakumari , R. A. Romano , R. G. Rosa , A. G. Salvio , V. Yakovlev , C. Kurachi , J. M. Hirshburg , J. A. Jo , Biomed. Opt. Express 2024, 15, 4557. 39346997 10.1364/BOE.523831 PMC11427192

[23] M. Kizilov , V. Yakovlev , V. Cheburkanov , S. Jung , in APS March Meeting Abstracts, Vol. 2025 2025, p. OD01–012.

[24] E. Duran‐Sierra , S. Cheng , R. Cuenca , B. Ahmed , J. Ji , V. V. Yakovlev , M. Martinez , M. Al‐Khalil , H. Al‐Enazi , Y.‐S. L. Cheng , J. Wright , C. Busso , J. A. Jo , Cancers 2021, 13, 4751. 34638237 10.3390/cancers13194751 PMC8507537

[25] M. S. Schmidt , P. Kennedy , G. Noojin , R. Thomas , B. Rockwell , J. Biomed. Opt. 2016, 21. 10.1117/1.JBO.21.1.015013 26818713

[26] W. Yi , M. Su , Y. Shi , S. Jiang , S.‐z. Xu , T. Lei , Cell Cycle 2018, 17, 844. 29623762 10.1080/15384101.2018.1456601 PMC6056223

[27] W. Hu , N. Mi , Y. Xu , G. Zhao , W. Gu , Lasers Med. Sci. 2020, 35, 1801. 32472428 10.1007/s10103-020-03012-3

[28] M. Luecking , R. Brinkmann , S. Ramos , W. Stork , N. Heussner , Comput. Biol. Med. 2020, 122, 103835. 32479348 10.1016/j.compbiomed.2020.103835

[29] K. Alghamdi , A. Kumar , A. Al‐ghamdi , A. Al‐rikabi , M. Mubarek , A. Ashour , Lasers Med. Sci. 2016, 31, 1819. 27572715 10.1007/s10103-016-2057-x

[30] A. Saha , R. Arora , V. V. Yakovlev , J. M. Burke , J. Biophotonics 2011, 4, 805. 21800432 10.1002/jbio.201100008

[31] V. V. Yakovlev , R. J. Thomas , G. Noojin , M. Denton , in Imaging, Manipulation, and Analysis of Biomolecules, Cells, and Tissues VI, Vol. 6859, SPIE 2008, pp. 90–97.

[32] K. Suhling , L. M. Hirvonen , J. A. Levitt , P.‐H. Chung , C. Tregidgo , A. Le Marois , D. A. Rusakov , K. Zheng , S. Ameer‐Beg , S. Poland , S. Coelho , R. Henderson , N. Krstajic , Med. Photonics 2015, 27, 3.

[33] V. Cheburkanov , M. Kizilov , V. Yakovlev , in Optical Diagnostics and Sensing XXV: Toward Point‐of‐Care Diagnostics, Vol. 13316, International Society for Optics and Photonics (Eds: G. L. Coté , J. S. Baba ), SPIE 2025, p. 133160K.

[34] H. Biswas , R. Tang , M. Michie , M. Kizilov , V. Cheburkanov , V. V. Yakovlev , M. Y. Berezin , in Reporters, Contrast Agents, and Molecular Probes for Biomedical Applications XVI, Vol. PC13339, International Society for Optics and Photonics (Eds: M. Y. Berezin , R. Raghavachari ) SPIE 2025, p. PC1333902.

[35] M. Kizilov , V. Yakovlev , V. Cheburkanov , S. Jung , in APS March Meeting Abstracts, Vol. 2025 2025, p. OD01–011.

[36] V. Yakovlev , Biochemical Applications Of Nonlinear Optical Spectroscopy, CRC Press 2018.

[37] M. Kizilov , S. Jung , V. Cheburkanov , V. Yakovlev , in Multimodal Biomedical Imaging XX, Vol. 13309, International Society for Optics and Photonics (Eds: X. Intes , M. Ochoa , M. A. Yaseen ) SPIE 2025, p. 133090L.

[38] Y. Shimojo , T. Nishimura , D. Tsuruta , T. Ozawa , H. H. L. Chan , T. Kono , Lasers Surg. Med. 2024, 56, 404. 38436524 10.1002/lsm.23773

[39] A. Kauvar , Semin. Cutaneous Med. Surg. 2012, 31, 126. 10.1016/j.sder.2012.02.002 22640433

[40] J. Harrington , V. Cheburkanov , M. Kizilov , I. Kulagin , G. Petrov , V. V. Yakovlev , Chem.‐Methods 2025.

[41] M. Kizilov , V. Cheburkanov , J. Harrington , V. Yakovlev , in Optical Biopsy XXIII: Toward Real‐Time Spectroscopic Imaging and Diagnosis, Vol. 13311, International Society for Optics and Photonics (Eds: R. R. Alfano , A. B. Seddon , L. Shi , B. Wu ) SPIE 2025, p. 133110F.

[42] R. Cubeddu , F. Docchio , R. Ramponi , M. Boulton , IEEE J. Quantum Electr. 1990, 26, 2218.

[43] J. T. Harrington , V. Cheburkanov , M. Kizilov , I. Kulagin , G. Petrov , V. V. Yakovlev , in Photonic Technologies in Plant and Agricultural Science II, Vol. 13357, International Society for Optics and Photonics, SPIE 2025, p. 1335702.

[44] R. K. Meleppat , K. E. Ronning , S. J. Karlen , M. E. Burns , E. N. Pugh Jr , R. J. Zawadzki ,Sci. Rep. 2021, 11, 16252. 34376700 10.1038/s41598-021-95320-z PMC8355111

[45] A. Dontsov , M. Ostrovsky , Int. J. Mol. Sci. 2024, 25, 3609. 38612421 10.3390/ijms25073609 PMC11011557

[46] S. Seidenari , F. Arginelli , C. Dunsby , P. French , K. König , C. Magnoni , C. Talbot , G. Ponti , PLoS ONE 2013, 8. 10.1371/journal.pone.0070682 PMC3724798 23923016

[47] F. Arginelli , M. Manfredini , S. Bassoli , C. Dunsby , P. French , K. König , C. Magnoni , G. Ponti , C. Talbot , S. Seidenari , Skin Res. Technol. 2013, 19. 10.1111/srt.12035 23279266

[48] C. Fink , M. Hofmann , A. Jagoda , I. Spaenkuch , A. Forschner , I. Tampouri , D. Lomberg , D. Leupold , C. Garbe , H. Haenssle , BMJ Open 2016, 6. 10.1136/bmjopen-2016-012730 PMC5168683 27993903

[49] M. T. Cone , J. D. Mason , E. Figueroa , B. H. Hokr , J. N. Bixler , C. C. Castellanos , G. D. Noojin , J. C. Wigle , B. A. Rockwell , V. V. Yakovlev , E. S. Fry , Optica 2015, 2, 162.

[50] G. Fürtjes , D. Reinecke , N. von Spreckelsen , A.‐K. Meißner , D. Rueß , M. Timmer , C. Freudiger , A. Ion‐Margineanu , F. Khalid , K. Watrinet , C. Mawrin , A. Chmyrov , R. Goldbrunner , O. T. Bruns , V. Neuschmelting , Front. Oncol. 2023, 13, 1146031. 37234975 10.3389/fonc.2023.1146031 PMC10207900

[51] A. E. Dontsov , R. D. Glickman , M. A. Ostrovsky , Free Radical Biol. Med. 1999, 26, 1436. 10401607 10.1016/s0891-5849(99)00003-9

[52] M. L. Denton , G. D. Noojin , M. S. Foltz , V. V. Yakovlev , L. E. Estlack , R. J. Thomas , B. A. Rockwell , J. Biomed. Opt. 2013, 18, 110501. 24193944 10.1117/1.JBO.18.11.110501

[53] J. Roegener , R. Brinkmann , C. P. Lin , J. Biomed. Opt. 2004, 9, 367. 15065904 10.1117/1.1646413

[54] A. Savitzky , M. J. Golay , Anal. Chem. 1964, 36, 1627.

[55] J. Ve č e ř, A. Kowalczyk , L. Davenport , R. Dale , Rev. Sci. Inst. 1993, 64, 3413.

[56] W. Becker , A. Bergmann , M. Hink , K. König , K. Benndorf , C. Biskup , Microscopy Res. Tech. 2004, 63, 58. 10.1002/jemt.10421 14677134

[57] K. V. Price , in Handbook of Optimization: From Classical to Modern Approach, Springer 2013, pp. 187–214.

[58] J. Kim , M. Lee , J. Phys. Chem. A 1999, 103, 3378.

[59] K. C. Elbert , Ph.D. thesis, University of Pennsylvania 2021.

[60] G. E. Uhlenbeck , L. S. Ornstein , Phy. Rev. 1930, 36, 823.

[61] M. Fazel , S. Jazani , L. Scipioni , A. Vallmitjana , E. Gratton , M. Digman , S. Pressé , ACS Photonics 2022, 9, 1015. 35847830 10.1021/acsphotonics.1c01936 PMC9278809

[62] L. Hirvonen , K. Suhling , Front. Phys. 2020, 8.

[63] M.‐J. Sun , Y.‐C. Zhang , F. Lin , S. Wang , L. Liu , J. Qu , APL Photonics 2024, 9.

[64] K. K. D. Tan , M. A. Tsuchida , J. Chacko , N. A. Gahm , K. Eliceiri , Front. Bioinform. 2023, 3. 10.3389/fbinf.2023.1286983 PMC10720713 38098814

[65] A. Pena , S. Ito , T. Bornschlögl , S. Brizion , K. Wakamatsu , S. Del Bino , Int. J. Mol. Sci. 2023, 24. 10.3390/ijms24054517 PMC10002570 36901948

[66] M. Kizilov , FLIM Reconvolution Toolkit, https://github.com/mkizilov/ReconFit.

[67] D. Herrera‐Ochoa , I. Llano , C. Ripoll , P. Cybulski , M. Kreuzer , S. Rocha , E. M. García‐Frutos , I. Bravo , A. Garzón‐Ruiz , J. Mater. Chem. B 2024, 12. 10.1039/d4tb00567h 38984432

[68] E. K. Ashworth , M.‐H. Kao , C. S. Anstöter , G. Riesco‐Llach , L. Blancafort , K. M. Solntsev , S. Meech , J. Verlet , J. Bull , Phys. Chem. Chem. Phys. 2023, 25. 10.1039/d3cp03250g 37649445

[69] T. Meyer , H. Bae , S. Hasse , J. Winter , T. Woedtke , M. Schmitt , K. Weltmann , J. Popp , Micromachines 2019, 10. 10.3390/mi10090564 PMC6780561 31454918

[70] F. Dake , Y. Taki , Appl. Opt. 2018, 57, 757. 29400744 10.1364/AO.57.000757

[71] S. Seidenari , F. Arginelli , C. Dunsby , P. French , K. König , C. Magnoni , C. Talbot , G. Ponti , PLoS ONE 2013, 8. 10.1371/journal.pone.0070682 PMC3724798 23923016

[72] Y. Ma , Y. Lee , C. Best‐Popescu , L. Gao , Proc. Natl. Acad. Sci. 2020, 118.

[73] J. Ryu , U. Kang , J. Kim , Biomed. Opt. Express 2018, 9, 3449. 29984109 10.1364/BOE.9.003449 PMC6033550

[74] M. e. a. Bénard , Int. J. Mol. Sci. 2021, 22.

