# Supplementary material for: Fluorescence Lifetime Imaging Microscopy Analysis of Isolated Melanosomes
Source: Chemphyschem. 2025 Jun 15;26(15):e202500034. doi: 10.1002/cphc.202500034 (PMC12321278; doi:10.1002/cphc.202500034)
Supplement: Supplementary file 1 — Supplementary Material [file CPHC-26-e202500034-s001.pdf]

Supplementary Table 1: Extracted Lifetime Components and Amplitude Ratios from 60 Time Points of Melanosome Imaging

	tau1	tau2	ampl1	ampl2
1	0.0012593849317574500	0.09481271666335180	14282.375818088100	98.1169378876829
2	0.00469834983408925	0.11967430134373200	6027.5443601142800	142.79640454580300
3	0.007377657156097160	0.13559869513600100	4498.505289288720	168.2115190628680
4	0.009166107761373630	0.1550729117939410	4080.1987957480000	182.88183645195100
5	0.01192026355898700	0.17306169288762000	3351.3101507690600	187.08042133635000
6	0.014116151114359700	0.18475963895133000	2682.310140739450	176.41211697244300
7	0.016295671289070900	0.1974015006209820	1775.914555825980	139.30804419505400
8	0.018734614755727400	0.22402250617687000	1154.1113879057300	101.47242572005300
9	0.020415602606276500	0.2428661858919090	837.5127068177640	78.56175455883650
10	0.022185980776621200	0.27008253520111800	709.6199940288390	62.967157594695700
11	0.027285258983004100	0.3711248443984120	489.05905172971000	40.49609410960360
12	0.029100313402334700	0.4140014012500850	353.9347111634280	30.05320575183700
13	0.029644641537984500	0.42252089614657800	296.9262871964550	26.41911762079970
14	0.034119398933114300	0.4178394589378980	95418.7405671847	8848.643260842230
15	0.030285241187816900	0.4536059185908300	237.03041591819800	21.617124366739400
16	0.030680217116310900	0.46680742437107900	198.0623175031210	18.805097238373500
17	0.029932414679508600	0.47353582862660300	187.65046318582400	17.894456920838600
18	0.032972787580875200	0.4902172314904890	159.4079097128610	15.970640748416400
19	0.03406490976713400	0.5007115406207020	144.12095002672600	14.638666913916800
20	0.028408080385016200	0.49334650663620100	145.96057301253000	14.496350123935800
21	0.031626691656859400	0.5047165110260790	131.17082394081100	13.969406575125500
22	0.032824881211114800	0.5312360713723600	117.98100302349400	12.142416250908800
23	0.03589311214227200	0.5690068844941720	96.80395156130270	10.18370465430040
24	0.0301023286933938	0.5475724292610130	97.29908966033510	10.518941327615600
25	0.037370873168992100	0.6110973679734800	76.99054397922310	8.723479333517560
26	0.03562088633049780	0.6003110402211800	72.95377507086900	8.763465774616690
27	0.034031183851745300	0.629926016165251	69.34012518942380	7.872715849144040
28	0.03619319429163700	0.645147898204651	60.109045248822600	7.226635859914890
29	0.03454079435815530	0.6479242912429600	57.29376537917540	7.130759626812340
30	0.040486697426357300	0.6692463404000520	49.38531943435980	6.53137089755323

	tau1	tau2	ampl1	ampl2
31	0.03148925697551950	0.6112525655168070	53.04544682046810	6.900959157856350
32	0.037577263824463000	0.7080356984004940	44.48138985905410	5.768852286564910
33	0.03270741553049090	0.6817938307357270	48.953838919084300	5.816824717015590
34	0.031400851322856300	0.6628049567060730	47.823949010473600	5.858085127226980
35	0.0376256542048545O	0.705830817024324	39.22968371155470	5.20305087337754
36	0.03243573845014470	0.4990217189407170	41.081578063477200	6.064494655888960
37	0.03516192101936640	0.6837574743033390	37.835751129792900	5.189615028428700
38	0.03966018758330870	0.7100596100945800	34.94841354221710	4.80715207738899
39	0.03377029817999560	0.7323817492620260	37.46601036380510	4.731218459595030
40	0.04074477634668820	0.7188495499611390	32.638256534900800	4.43359121797633
41	0.036902896325276300	0.7076305356794690	31.07708516498090	4.52844690580822
42	0.03900770781629200	0.7140359438240060	29.594842333780300	4.399044298822180
43	0.03142826448978480	0.688022589400995	31.710810446858000	4.388969913018320
44	0.04056778022134640	0.785218187414219	27.669448952065300	3.7827613351340900
45	0.03858107834341270	0.7751861949252010	27.587304923134700	3.9647928398448600
46	0.04555278329188180	0.7358463985544710	23.819666649436500	3.9069196138758800
47	0.03874578676750250	0.7698531755273870	26.12699918461080	3.9227632816495800
48	0.03984953240308480	0.7943603635785600	26.150193402249700	3.706620953422050
49	0.03944340577842230	0.7790862818150410	25.21009550088710	3.6135892303650500
50	0.037406946607269100	0.7934995762352310	25.67357178470390	3.515812435861440
51	0.03990251806121960	0.6516502642015750	23.684809901462100	3.7879440715217800
52	0.03037491751405030	0.7203145906344970	28.303375884228700	3.916878225625260
53	0.032555452214853400	0.7423088423826800	27.351808901130900	3.724877854347790
54	0.030938287417381500	0.725245264431829	27.91122127327330	3.794566327176580
55	0.0293211226199096	0.7081816864809790	28.470633645415600	3.864254800005360
56	0.03021509182981660	0.5715454265646530	27.30462962183170	3.9439487962898200
57	0.034910920890867100	0.7512749131110000	25.48714221524980	3.5497003431835300
58	0.03438741066966570	0.7633043501267760	26.284497784081000	3.626954813811690
59	0.025785593746908300	0.49324216780028500	29.536908704981000	4.501004045310050
60	0.04135428173361760	0.7800485073269410	24.306370494626900	3.344940448775840
